# Multi-Objective Optimization of Ultrasonic Surface Rolling Process Parameters for TC4 Titanium Alloy with IWOA–RBF and MOGWO Algorithms

**DOI:** 10.3390/mi17040451

**Published:** 2026-04-06

**Authors:** Yeshen Lan, Chuchu Rao, Yunpeng Lyu

**Affiliations:** 1 School of Mechanical Engineering, Quzhou College of Technology, Quzhou 324000, China; yelance0331@163.com (Y.L.);; 2 College of Mechanical Engineering, Zhejiang University of Technology, Hangzhou 310023, China; 3 College of Engineering, University of Missouri, Columbia, MO 65211, USA

**Keywords:** USRP, IWOA-RBF prediction model, MOGWO, TC4 titanium alloy, process parameter optimization

## Abstract

A structured optimization approach was applied to ultrasonic surface rolling process (USRP) parameters, aiming to enhance the material surface characteristics of TC4 titanium alloy. To overcome the premature convergence and limited exploration capability of the standard Whale Optimization Algorithm (WOA), three enhancement strategies were introduced, including population initialization based on an optimal point set, a sinusoidal nonlinear convergence factor, and an adaptive inertia-based position update strategy. By optimizing the structural parameters of the RBF neural network with the improved WOA, an IWOA–RBF predictive model for surface performance evaluation was developed and rigorously validated in terms of prediction accuracy. Using the developed IWOA–RBF model, a multi-criteria decision-making framework integrating the CRITIC weighting method and the TOPSIS ranking approach was constructed to evaluate surface quality. This framework was further combined with a multi-objective Grey Wolf Optimization (MOGWO) algorithm to perform Pareto-based optimization and determine the optimal USRP parameter set. Experimental validation showed that the optimized parameters resulted in a significant reduction in surface roughness, while enhancing both surface hardness and residual compressive stress. The results confirm the robustness and effectiveness of the proposed IWOA–RBF and MOGWO optimization framework, providing a reliable strategy for high-precision parameter optimization and coordinated enhancement of surface properties in the TC4 titanium alloy USRP.

## 1. Introduction

TC4 titanium alloy [1,2,3] is widely used in aerospace applications due to its excellent specific strength, exceptional corrosion resistance, and superior mechanical properties at high temperatures. Nevertheless, when titanium alloy components operate under harsh and complex service environments, they are prone to surface-related degradation phenomena, such as fatigue damage and wear [4,5], which can markedly shorten their service life and compromise operational reliability [6,7,8]. USRP [9,10,11] is an advanced hybrid surface strengthening method that integrates high-frequency ultrasonic vibration with static rolling force to promote severe plastic deformation within the near-surface layer [12]. Through this mechanism, surface roughness is significantly reduced, surface microstructures are refined, and favorable residual compressive stress distributions are introduced, thereby enhancing fatigue performance and overall surface integrity [13,14,15]. In comparison with conventional surface modification techniques [16,17,18,19], USRP offers improved controllability and greater flexibility for surface integrity optimization, advantages that are primarily attributed to its vibration-assisted deformation characteristics.

The performance of USRP is strongly governed by the accurate regulation of its process parameters. Numerous studies [20,21] have confirmed that parameter variations exert a pronounced influence on surface morphology, residual stress characteristics, and microstructural evolution. For example, Su et al. [22] and Wang et al. [23] independently identified feed rate as a key factor affecting surface quality of GCr15 and M50 steels. Liu et al. [24] reported that, when applied to 7075-T6 aluminum alloy, higher static pressure enhances gradient hardening and deepens the compressive stress field. Pang et al. [25] highlighted the significance of spindle speed in determining fatigue behavior during ultrasonic rolling of Ti-6Al-4V alloy. In addition, Zha et al. [26] demonstrated that higher ultrasonic amplitude leads to increased impact kinetic energy and strain rate, resulting in more pronounced surface strengthening. Tong et al. [27] further revealed that strong coupling effects exist among the process parameters, jointly governing the material surface characteristics of TC4 titanium alloy.

Despite these studies, the relationship linking USRP parameters to processing quality remains highly nonlinear and is governed by strong parameter coupling. Traditional optimization strategies, including orthogonal experimental design [28] and response surface methodology [29], often suffer from limited global search capability and reduced computational efficiency when dealing with multivariable and multi-objective optimization tasks. By contrast, artificial neural networks (ANNs) [30,31], owing to their powerful nonlinear modeling capacity and self-learning capability, have proven effective for describing complex manufacturing processes. Among various ANN models, RBF neural networks [32,33,34] have been widely applied in machining time estimation, energy consumption prediction, and process optimization [35,36,37], benefiting from their relatively simple structure and fast convergence. Nevertheless, the prediction capability of RBF neural networks is strongly influenced by the selection of their internal parameters. When tuned using conventional empirical or gradient-based methods, RBF neural networks are prone to premature convergence, which constrains their applicability in high-precision surface strengthening processes such as USRP.

Owing to its concise algorithmic structure, limited number of control parameters, and strong global exploration capability [38,39], WOA has been utilized successfully in a wide spectrum of optimization problems [40,41,42,43,44]. In recent years, WOA has frequently been combined with data-driven predictive models, including backpropagation (BP) neural networks [45] and extreme gradient boosting (XGBoost) models [46,47], leading to notable improvements in process modeling and optimization performance [48,49]. Nevertheless, when applied to challenging optimization problems, WOA often suffers from drawbacks such as slow convergence and premature convergence [50,51]. To overcome these issues, Mezaache et al. [52] introduced an inertia-weight-based WOA variant, which demonstrated enhanced convergence characteristics and superior solution quality in welding process optimization. Building upon this line of research, Ye et al. [53] further proposed an improved WOA and developed an IWOA–ANN predictive framework, achieving substantial gains in prediction accuracy and algorithm robustness. Collectively, these studies provide valuable guidance for improving the effectiveness of WOA in complex process modeling and optimization tasks.

Inspired by the aforementioned studies, the present work developed a hybrid optimization framework that integrates an IWOA–RBF model with the MOGWO to optimize the USRP parameters of TC4 titanium alloy. Initially, the WOA was systematically refined through improvements in population initialization, convergence control, and position update strategies, leading to improved exploration capability and convergence performance. Subsequently, the improved WOA was used to fine-tune the critical parameters of the RBF neural network, resulting in a robust IWOA–RBF predictive model. The coupling of the trained IWOA–RBF model with the MOGWO algorithm enables the Pareto-based multi-objective optimization of the surface performance indicators. To determine the most suitable compromise solution from the obtained Pareto front, an integrated CRITIC–TOPSIS decision-making approach is adopted. Finally, experimental validation of the optimized parameter combination was performed, confirming the effectiveness and reliability of the proposed framework. This study presents a structured approach to optimize USRP parameters and improve the surface integrity of TC4 titanium alloy.

## 2. Materials and Methods

### 2.1. Experimental Procedure

**Figure 1:**
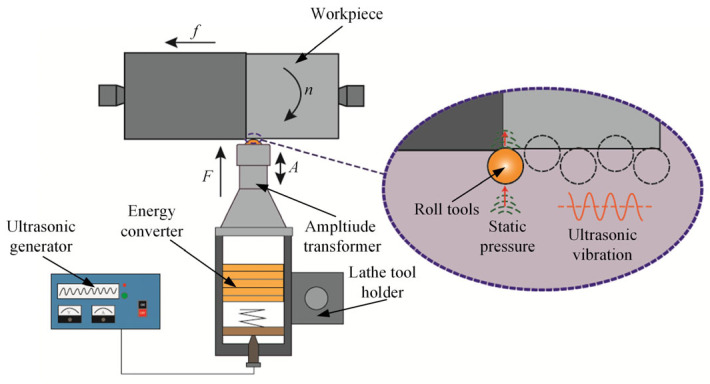
Schematic illustration of the USRP.

The experiments employed TC4 titanium alloy in the form of cylindrical bars (50 mm × 320 mm). Prior to USRP, all specimens were subjected to turning operations to minimize cylindricity errors. After machining, the surface properties were characterized by a surface roughness (*R_a_*) of 0.862 μm, hardness (*H_v_*) of 229.7 HV, and residual compressive stress (*σ_res_*) of −126 MPa. USRP is a high-efficiency and precision surface strengthening technique that operates without material removal based on high-frequency ultrasonic vibration, as schematically illustrated in Figure 1. In this configuration, ultrasonic electrical signals generated by the power supply are converted into mechanical vibrations through the transducer, then amplified via the horn, and finally directed to the rolling tool head as high-frequency oscillations. By combining ultrasonic vibration with static rolling pressure, the tool head continuously interacts with the workpiece surface, inducing localized plastic deformation and enhancing surface integrity.

*R_a_* was measured using a Mitutoyo SJ-210 roughness tester (Mitutoyo Corporation, Kawasaki, Japan), while *H_v_* was evaluated with an HVS-1000A microhardness tester (Zhongte, Dongguan, China) under a load of 300 gf and a dwell time of 10 s. *σ_res_* on the processed surface was measured with an Xstress G2R X-ray stress analyzer (Stresstech Oy, Helsinki, Finland). To enhance the reliability and repeatability of the experimental results, each reported value represents the arithmetic mean of measurements obtained from three randomly selected positions on the specimen surface.

### 2.2. Experimental Design and Results

Four primary USRP parameters were selected for investigation: spindle speed (*n*), feed rate (*f*), static pressure (*F*), and amplitude (*A*). The specific ranges and levels for these parameters were determined based on preliminary exploratory experiments, which were designed to cover a wide range of values in order to capture their influence on the process. Each parameter was investigated across five different levels, as listed in Table 1. Based on the selected factor levels, an orthogonal experimental scheme was designed. To ensure statistical reliability and assess process stability, each experimental trial was repeated three times under identical conditions. For each parameter combination, the mean values and standard deviations of the surface performance indicators (*R_a_*, *H_v_*, and *σ_res_*) were calculated. Table 2 presents the experimental results for each parameter combination.

## 3. IWOA-RBF Neural Network Prediction Model

### 3.1. RBF Neural Networks

**Figure 2:**
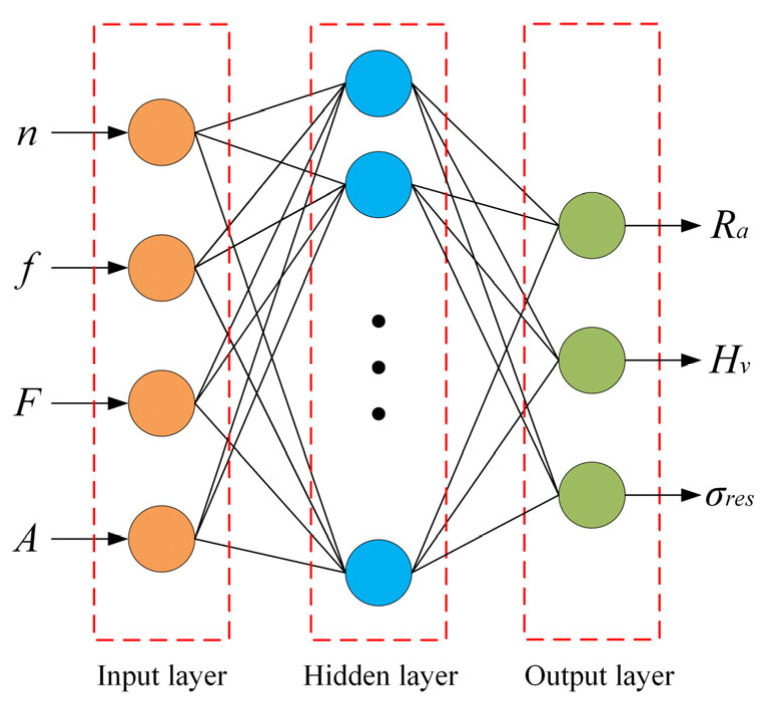
Architecture of the RBF neural network.

As shown in Figure 2, the input layer of the RBF neural network is constructed using the four USRP parameters defined in Section 2.2, while the output layer corresponds to the surface performance indicators. Accordingly, each input sample can be expressed as a vector *L* = (*l*_1_, *l*_2_, *l*_3_, *l*_4_). The hidden layer is composed of a set of RBF neurons, the number of which is determined according to the size of the training dataset and the desired generalization performance of the network. Each hidden neuron is characterized by a radial basis center vector *C_i_* = (*C_i_*_1_, *C_i_*_2_, *C_i_*_3_, *C_i_*_4_). The corresponding mathematical formulation is expressed as follows:

(1) $$y\left(x\right)=\sum_{i=1}^{N}\omega_{i}\left(\frac{-{\left\|L-C_{i}\right\|}^{2}}{2\sigma_{i}^{2}}\right)$$

where *N* represents the size of the hidden layer, *σ_i_* controls the width of the *i*-th RBF, and *ω_i_* corresponds to the weights connecting the hidden and output layers.

### 3.2. Improved WAO for RBF Optimization

For the prediction of surface properties for TC4 titanium alloy subjected to USRP, the optimization of key RBF neural network parameters (*ω_i_*, *σ_i_*, and *C_i_*), as defined in the previous section, is essential for achieving high prediction accuracy and robust generalization performance. Traditional parameter optimization methods based on stochastic gradient schemes are highly dependent on initial parameter settings and often suffer from premature convergence when handling strongly nonlinear relationships, which in turn degrades prediction robustness. To overcome these drawbacks, the improved WOA is adopted to globally optimize the RBF network parameters.

#### 3.2.1. Population Initialization Strategy Based on Optimal Point Set

In the WOA, population individuals are typically generated using random initialization, which often results in uneven distribution of candidate solutions within the search space and may consequently impair global exploration efficiency. To address this issue, an initialization strategy based on an optimal point set is employed in this study to construct the initial population, thereby enhancing the uniformity of solution space coverage. Let *n* denote the population size and *d* denote the dimensionality of the search space. The position of the *i*-th whale individual can be represented as

(2) $$X_{i}=\left(x_{i1},x_{i2},\cdots ,x_{id}\right),i=1,2,\cdots ,n$$

Based on the optimal point set, the population initialization process can be formulated as

(3) $$\left\{\begin{array}{c} P_{ij}=\left\{r_{i1},\cdots ,r_{ij},\cdots ,r_{id}\right\}\\ r_{j}=e^{j}\\ x_{ij}\left(o\right)=x_{lbj}+\left(x_{ubj}-x_{lbj}\right)\cdot p_{ij} \end{array}\right.$$

where *P_i_* denotes the optimal point set corresponding to the *i*-th whale; *r_ij_* represents the element of the optimal point set of the *i*-th whale in the *j*-th dimension, where *j* = 1, 2, …, *d*; and *x_ubj_* and *x_lbj_* specify the feasible search interval of that dimension.

**Figure 3:**
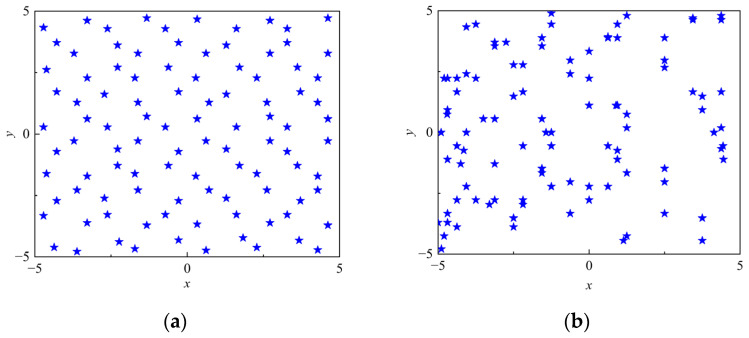
Comparison of population initialization methods: (**a**) optimal point set initialization and (**b**) random initialization.

A two-dimensional test case (*n* = 100, *d* = 2) is considered to evaluate the proposed initialization strategy, and the upper and lower bounds are defined as *x_ub_* = [5, 5] and *x_lb_* = [−5, −5], respectively. Figure 3 compares the population distributions generated by random initialization and those obtained using the optimal point set-based strategy. The latter leads to more balanced coverage of the search space, alleviating clustering of solutions. Such an improved initial population distribution enhances global exploration capability, especially in the early optimization stages.

#### 3.2.2. Design of a Sinusoidal Nonlinear Convergence Factor

A linearly decreasing convergence factor *a* is used in the WOA. While this linear attenuation scheme facilitates global exploration during the initial search phase, it may lead to suboptimal convergence efficiency. Moreover, in the later stages of optimization, an excessively small convergence factor tends to diminish the algorithm’s local exploitation capability, thereby limiting further refinement of candidate solutions. To address these shortcomings, a sinusoidal-based nonlinear decay strategy is introduced for the convergence factor, denoted as *a*_1_, as expressed in Equation (4).

(4) $$a_{1}=\left(a_{\max}-a_{\min}\right)\cdot \left(1-\sin\left({\left(t/T\right)}^{2}\cdot \frac{\pi }{2}\right)\right)$$

The convergence behavior of the proposed sinusoidal nonlinear convergence factor is systematically contrasted with that of several alternative decay strategies. The corresponding convergence factors, denoted as *a*_2_, *a*_3_, *a*_4_, and *a*_5_, are defined in Equation (5).

(5) $$\left\{\begin{array}{c} a_{2}=a_{\max}-\left(a_{\max}-a_{\min}\right)\cdot \frac{t}{T}\\ a_{3}=a_{\max}-\left(a_{\max}-a_{\min}\right)\cdot \frac{1}{e-1}\cdot \left(e^{\frac{t}{T}}-1\right)\\ a_{4}=\left(a_{\max}-a_{\min}\right)\cdot \cos\left(\frac{t}{T}\right)\\ a_{5}=a_{\max}-\left(a_{\max}-a_{\min}\right)\cdot {\left(\frac{t}{T}\right)}^{2} \end{array}\right.$$

where *a*_max_ and *a*_min_ specify the bounds of the convergence factor; *t* and *T* correspond to the current and total iteration numbers, respectively.

**Figure 4:**
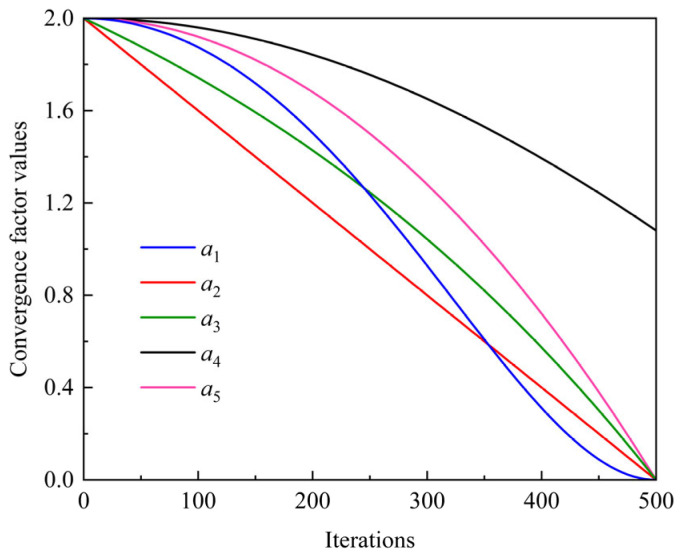
Evolution characteristics of different convergence factor strategies.

Figure 4 illustrates the comparative simulation results of the five convergence factor strategies. It can be observed that the sinusoidal nonlinear convergence factor preserves relatively larger values during the early iterations, which effectively enhances global exploration. As the search progresses, the convergence factor decreases in a smooth and gradual manner, thereby strengthening local exploitation and enabling more refined exploration in the neighborhood of high-quality solutions.

#### 3.2.3. Adaptive Inertia-Based Position Update Strategy

The WOA frequently exhibits limited convergence efficiency during the early stages of the search process and is susceptible to entrapment in local optima. As the optimization proceeds into later stages, population individuals tend to cluster rapidly around the current best solution, which reduces population diversity and increases the likelihood of premature convergence. To overcome these deficiencies, the position update rule is augmented with an inertia weight coefficient (*γ*), allowing adaptive regulation of the search process across different optimization phases.

When $\left|H\right|$ > 1,

(6) $$X_{k(t+1)}=X_{rt}-H\cdot \left|C\cdot X_{rt}-X_{kt}\right|$$

When $\left|H\right|$ ≤ 1,

(7) $$\left\{\begin{array}{c} X_{k(t+1)}=\gamma \cdot X_{bt}-H\cdot \left|C\cdot X_{bt}-X_{kt}\right|,p<0.5\\ X_{k(t+1)}=\gamma \cdot X_{bt}+\left|X_{bt}-X_{kt}\right|\cdot e^{ZQ}\cdot \cos\left(2\pi Q\right),p\geq 0.5 \end{array}\right.$$

where *X_kt_* represents the current position, *X_rt_* acts as a randomly sampled reference from the population, *X_bt_* signifies the current optimal position, *Z* describes the spiral search behavior, and *Q* is a random coefficient bounded within [−1, 1].

The step size of individual position updates is modulated by the *γ*, which is adaptively adjusted according to the following formulation:

(8) $$\gamma =\gamma_{1}+\left(\gamma_{1}-\gamma_{2}\right)\cdot \frac{2}{\pi }\cdot \arccos\frac{t}{T_{\max}}$$

where *γ*_1_ and *γ*_2_ represent the initial weight and final weight, respectively.

By employing this nonlinear adaptive weighting strategy, the improved WOA achieves a more balanced search behavior. Specifically, larger inertia weights in the early optimization stage encourage extensive exploration of the solution space, while progressively reduced weights in the later stage guide the search toward promising regions, thereby enhancing local exploitation and solution refinement.

**Figure 5:**
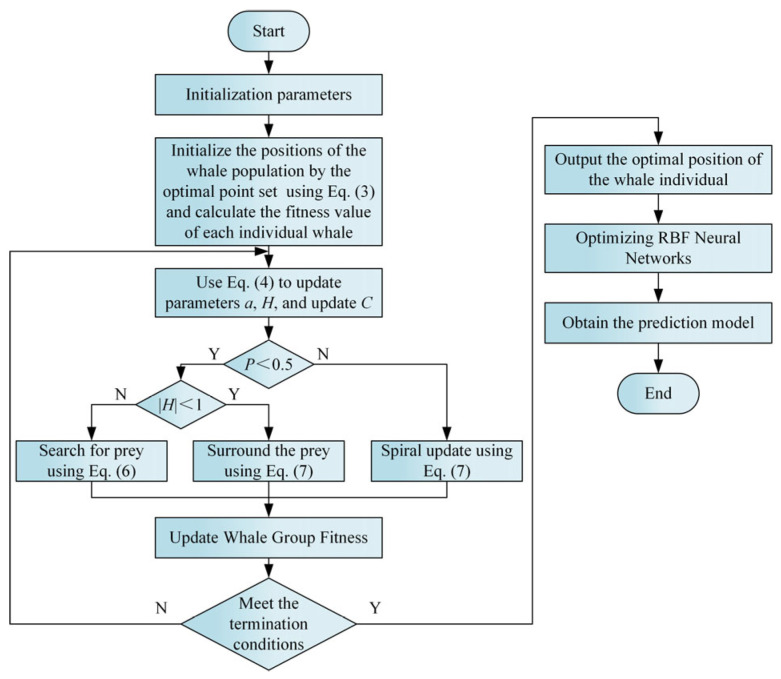
Flowchart of the IWOA–RBF neural network model for surface property prediction.

Building upon this enhancement, the WOA is further refined by integrating optimal point set-based population initialization, a sinusoidal nonlinear convergence factor, and the adaptive inertia-based position update strategy described above. Building on this, an IWOA–RBF neural network model is constructed to predict the *R_a_*, *H_v_*, and *σ_res_* of TC4 titanium alloy subjected to USRP. The overall algorithmic framework of the proposed approach is illustrated in Figure 5.

### 3.3. Prediction Model Results and Analysis

**Figure 6:**
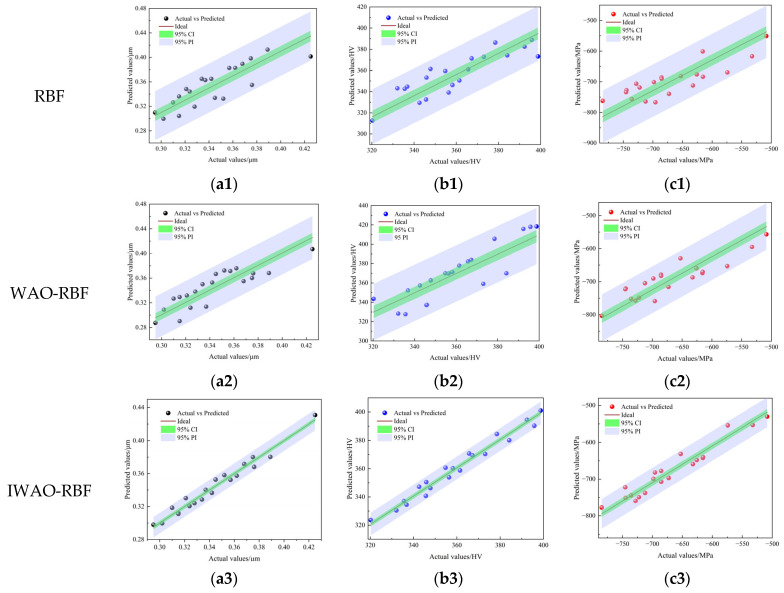
Predicted vs. actual values on the training dataset: (**a1**–**a3**) *R_a_*, (**b1**–**b3**) *H_v_*, and (**c1**–**c3**) *σ_res_*.

**Figure 7:**
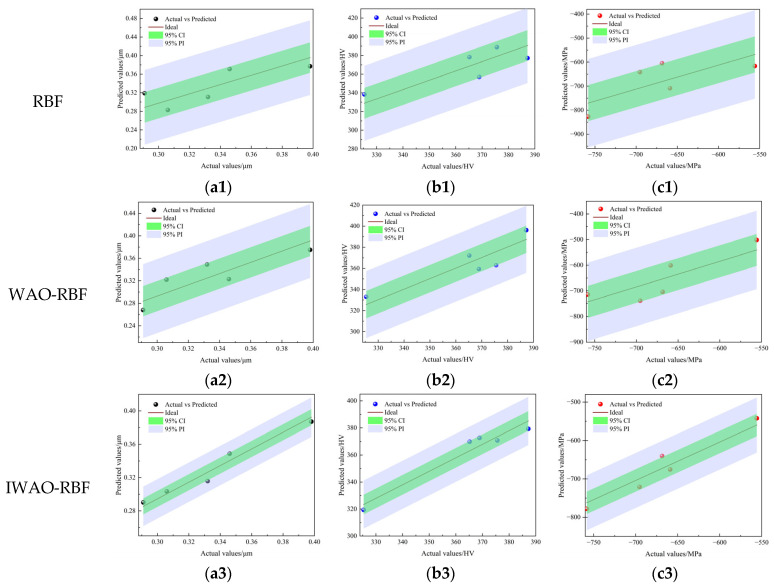
Predicted vs. actual values on the testing dataset: (**a1**–**a3**) *R_a_*, (**b1**–**b3**) *H_v_*, and (**c1**–**c3**) *σ_res_*.

The dataset presented in Table 2 was used for training and validation of the model. The data were randomly split into 20 samples for training and 5 for testing to validate predictive reliability. Balancing approximation accuracy and generalization depends on the number of hidden neurons, as excessively large or small configurations may lead to overfitting or underfitting, respectively. Based on empirical guidelines and iterative tuning, a hidden layer comprising 11 neurons was adopted. The optimization algorithm was implemented with a population of 100 individuals and an iteration limit of 200. Figure 6 and Figure 7 illustrate the predictive performance of the various models on the training and testing datasets, respectively. Both figures show the 95% confidence interval (CI) and the 95% prediction interval (PI) for each model. Compared to the RBF and WOA-RBF models, the IWOA-RBF model exhibits the smallest deviation between predicted and actual values, with its confidence and prediction intervals being notably narrower. In Figure 6 (training dataset), the predicted points from the IWOA-RBF model are closely aligned with the ideal line, indicating strong fitting performance. In Figure 7 (testing dataset), the predicted values exhibit minimal dispersion, and all test samples fall within the 95% PI, highlighting the model’s superior generalization capability and low prediction uncertainty.

To further assess the prediction accuracy of each model, the RMSE, MAPE, and R^2^ were used to evaluate model performance on the testing dataset. The corresponding results are summarized in Table 3. The IWOA-RBF model consistently outperformed both the RBF and WOA-RBF models across all evaluation metrics. Specifically, for *R_a_*, the IWOA-RBF model achieved an RMSE of 0.0060 μm, a MAPE of 1.34%, and an R^2^ of 0.9603 on the testing dataset. For *H_v_*, the model achieved an RMSE of 6.39 HV, a MAPE of 1.71%, and an R^2^ of 0.9421. For *σ_res_*, despite the inherent challenges in prediction, the IWOA-RBF model still yielded the lowest RMSE (20.01 MPa) and MAPE (2.92%), along with the highest R^2^ (0.9294). These results demonstrate that the IWOA-RBF model more accurately represents the complex nonlinear interactions between USRP parameters and the surface properties of TC4 titanium alloy.

Table 4 compares the half-widths of the 95% CI and PI for each model. As shown in Figure 6 and Figure 7, the IWOA-RBF model consistently exhibits the narrowest confidence and prediction intervals across the training and testing datasets. For instance, in the testing dataset, the half-widths of the prediction intervals for the IWOA-RBF model are ±0.0237 μm (*R_a_*), ±17.82 HV (*H_v_*), and ±71.70 MPa (*σ_res_*), all of which are notably narrower than those of the RBF and WOA-RBF models. These results demonstrate that the IWOA-RBF model not only achieves higher prediction accuracy for the target performance indicators but also provides more stable and reliable predictions, further emphasizing its superiority in uncertainty quantification.

Although the IWOA-RBF model demonstrates strong overall predictive performance for the process parameters and surface properties of TC4 titanium alloy in the USRP, some limitations persist. Specifically, the model shows slightly lower prediction accuracy for *σ_res_* compared to *R_a_* and *H_v_*, as evidenced by a lower R^2^ value and relatively wider prediction intervals. This discrepancy is primarily attributed to the inherently higher variability of *σ_res_* measurements compared to *R_a_* and *H_v_* (as reflected by the standard deviations reported in Table 2). The greater absolute fluctuation in *σ_res_* data leads to a lower signal-to-noise ratio, increasing the difficulty for the model to capture the underlying physical relationships. Consequently, the R^2^ for *σ_res_*, while still indicating strong predictive capability, is slightly lower than those for *R_a_* and *H_v_*. Furthermore, the limited sample size may contribute to overfitting, potentially causing the model to learn noise rather than the true data patterns, which in turn can undermine its generalization capability. To mitigate these issues, future research will focus on expanding the training dataset both in terms of scale and diversity, utilizing techniques such as data augmentation and transfer learning to improve model robustness and generalization performance.

## 4. Process Parameter Optimization

### 4.1. Solution of Multi-Objective Optimization Models Based on MOGWO

The optimization of USRP parameters for TC4 titanium alloy constitutes a typical multi-objective optimization problem, in which the optimal solution is generally non-unique. Accordingly, identifying a Pareto-optimal solution ensemble that balances competing objectives becomes essential. To this end, the MOGWO, an extension of the GWO, is adopted to solve the proposed optimization problem.

During each iteration, MOGWO evaluates the fitness of individual grey wolves and retains the resulting non-dominated candidates in an external archive. In the archive management step, archive occupancy is examined against the predefined capacity. When this condition is met, an adaptive grid-based strategy is employed to partition the objective space and detect densely populated regions. One solution from the most crowded region is removed, while a newly generated solution is introduced into a sparsely populated region, thereby preserving diversity along the Pareto front.

**Figure 8:**
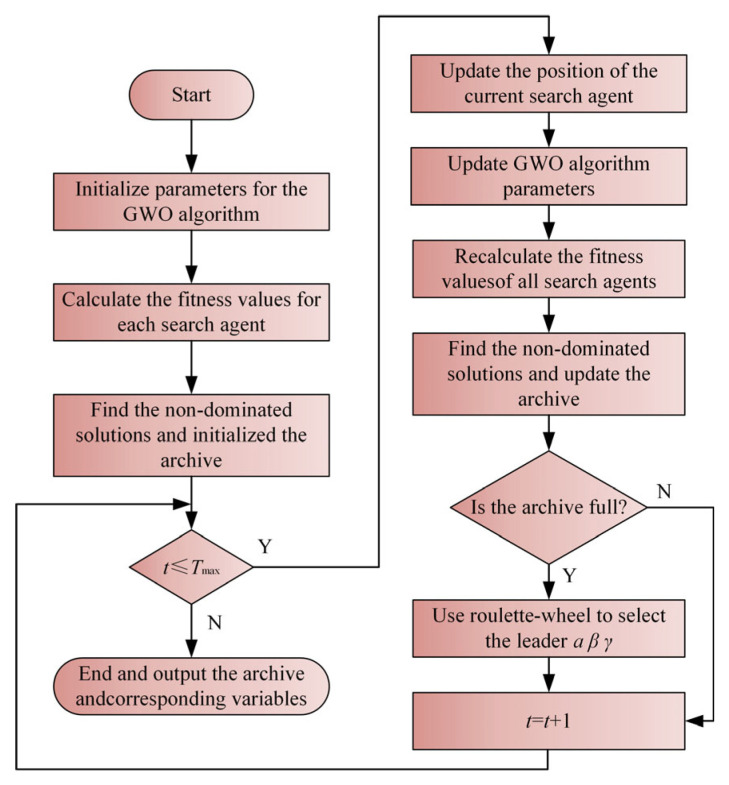
Flowchart of the MOGWO algorithm.

To guide the evolutionary process, three leader wolves (*α*, *β*, and *γ*) are chosen from the archive via a roulette-wheel-based strategy. The leader positions are used to steer the position evolution of the population. Upon completion of the iterative search, the non-dominated solutions preserved in the external archive constitute the final Pareto front. The overall procedure of the MOGWO algorithm is illustrated in Figure 8.

The USRP parameter optimization problem is formulated by considering *R_a_*, *H_v_*, and *σ_res_* as objective functions. From a performance perspective, a lower *R_a_* value corresponds to improved surface quality and is therefore treated as a minimization objective. In contrast, higher *H_v_* value is associated with enhanced wear resistance and is considered a maximization objective. Additionally, a lower magnitude of *σ_res_* value after USRP is preferred, and this objective is likewise minimized. Based on these considerations, the USRP is formulated as a multi-objective optimization problem, as given below.

(9) $$\left\{\begin{array}{c} \min y_{1}\left(n,f,A,F\right)\\ \max y_{2}\left(n,f,A,F\right)\\ \min y_{3}\left(n,f,A,F\right) \end{array}\right.$$

where *y*_1_, *y*_2_, and *y*_3_ are the normalized values for *R_a_*, *H_v_*, and *σ_res_*, respectively.

The constraints of the objective function are shown in Equation (10).

(10) $$s.t.\left\{\begin{array}{c} 150\leq n\leq 550\\ 0.1\leq f\leq 0.3\\ 250\leq F\leq 650\\ 3\leq A\leq 15 \end{array}\right.$$

### 4.2. Optimization Results and Validation

To ensure the reliability of the multi-objective optimization process, we first optimized the parameters of the MOGWO algorithm through a series of preliminary experiments. In these experiments, we evaluated both the convergence behavior and the diversity of the Pareto front, testing various combinations of population sizes (50, 100, 150), iteration counts (100, 200, 300), and archive sizes (30, 40, 50). The results indicated that a population size of 100, an archive size of 40, and 200 iterations provided the optimal trade-off between solution quality and computational efficiency. Based on these findings, these parameter settings were selected for the final optimization runs.

To avoid subjective bias in the selection of the optimal parameter combination, a comprehensive evaluation of the Pareto-optimal solutions was conducted based on *R_a_*, *H_v_*, and *σ_res_*. Accordingly, a hybrid CRITIC–TOPSIS decision-making approach was employed. The non-dominated solution set obtained from MOGWO was then evaluated and ranked using this framework.

The TOPSIS method evaluates the relative quality of candidate solutions by calculating their closeness to the ideal solution. Prior to the proximity assessment, all performance indicators were normalized to satisfy the requirements of the TOPSIS procedure. In this study, *R_a_* and *σ_res_* are treated as minimization objectives and classified as cost-type indicators, which are normalized using Equation (11). In contrast, *H_v_* is considered a maximization objective and categorized as a benefit-type indicator, which is normalized according to Equation (12):

(11) $$S_{ij}=\frac{\max\left(g_{ij}\right)-g_{ij}}{\max\left(g_{ij}\right)-\min\left(g_{ij}\right)}$$

(12) $$S_{ij}=\frac{g_{ij}-\min\left(g_{ij}\right)}{\max\left(g_{ij}\right)-\min\left(g_{ij}\right)}$$

Here, *g_ij_* represents the *j*-th evaluation indicator for the *i*-th solution group, where *i* = 1, 2, …, 40, and *j* = 1, 2, 3, *G* = [*g_ij_*]*_k_*_×*m*_ is the matrix formed from the Pareto solution set obtained via the MOGWO algorithm, which is then standardized to create the *S* matrix.

The CRITIC method calculates objective weights by incorporating both indicator dispersion and inter-criteria correlation (*ζ_j_*). Dispersion is expressed by the standard deviation (*σ_j_*), while *ζ_j_* is characterized through Pearson correlation analysis:

(13) $$\zeta_{j}=\sum_{i=1}^{k}\left(1-\rho_{ij}\right)$$

where *ρ_ij_* represents the Pearson correlation coefficient between evaluation indicators.

By integrating the effects of contrast intensity and inter-criteria conflict, the weights of the optimization objectives are calculated according to Equation (14).

(14) $$W_{j}=\frac{\sigma_{j}\times \zeta_{j}}{\sum_{j=1}^{m}\left(\sigma_{j}\times \zeta_{j}\right)}$$

**Figure 9:**
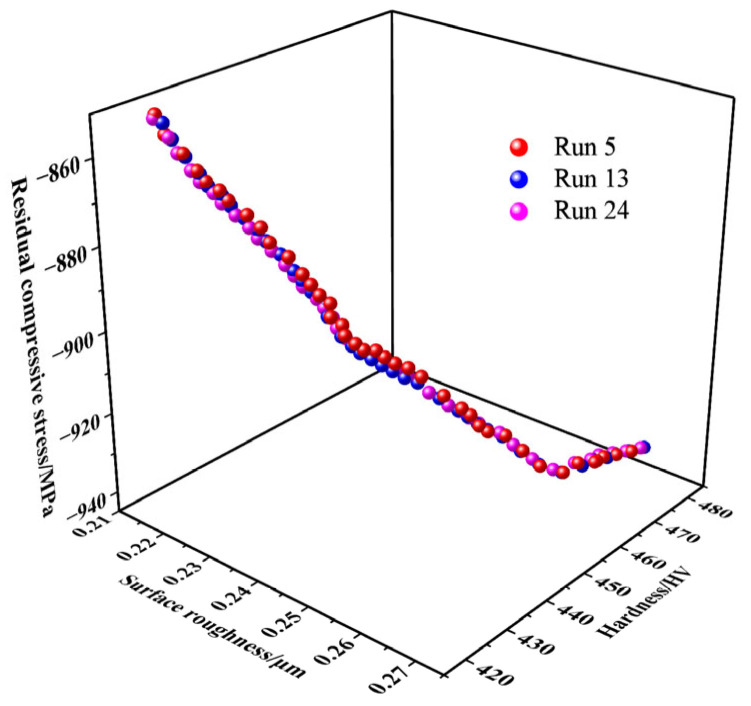
Pareto-optimal solution set obtained by the MOGWO algorithm.

The results of 30 independent MOGWO optimization runs are shown in Figure 9, which illustrates the Pareto front solutions obtained from three representative runs. As depicted, the reproducibility of the Pareto front across the independent runs is consistent, with minimal fluctuation. This suggests that the MOGWO algorithm delivers reliable and stable solutions across multiple optimization runs.

To further evaluate the Pareto-optimal solutions, the CRITIC–TOPSIS method was applied to rank the 40 solutions from each of the 30 independent runs. The ranking was based on closeness coefficients, calculated using the Euclidean distance between each candidate solution and the ideal solution. The highest-ranked solution from each run was selected for further analysis. Table 5 presents a statistical summary of the 30 top-ranked solutions, corresponding to the optimal solution of each independent run. The small standard deviations for both the four USRP process parameters and the three response variables indicate that the MOGWO algorithm exhibits good stability and consistency in optimizing the USRP for TC4 titanium alloy. Thus, the mean values of *n* = 410.3 r/min, *f* = 0.14 mm/r, *F* = 373.4 N, and *A* = 11.6 μm were selected as the final optimization results for subsequent experimental validation. The USRP equipment employed in this study offers high-resolution control capabilities, with programmable precision of 0.1 r/min for *n*, 0.01 mm/r for *f*, 0.1 N for *F*, and 0.1 μm for *A*. The theoretically optimized parameters—such as the representative solution sets obtained from the MOGWO algorithm—are summarized statistically in Table 5. The alignment between algorithmic precision and hardware resolution ensures that the optimization results are fully realizable in practice, thereby bridging the gap between theoretical optimization and practical manufacturing.

**Figure 10:**
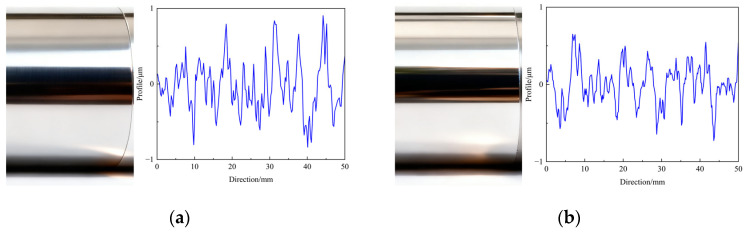
Surface morphologies and contour profiles of specimens processed under different parameter combinations: (**a**) control group and (**b**) optimized group.

To further examine the effectiveness of the optimized parameters, the TOPSIS–CRITIC decision-making approach was also applied to the orthogonal experimental data presented in Table 2. Based on the ranking results, the 14th experimental group (*n* = 350 r/min, *f* = 0.25 mm/r, *F* = 250 N, and *A* = 9 μm) was chosen as the control group for comparison. Subsequently, USRP tests were performed under both the optimized parameter set and the control group (the 14th group in Table 2). The resulting surface morphologies and contour profiles are shown in Figure 10. Compared with the control group, specimens processed under the optimized parameters exhibit noticeably smoother surface profiles, characterized by fewer surface grooves and reduced surface roughness.

**Figure 11:**
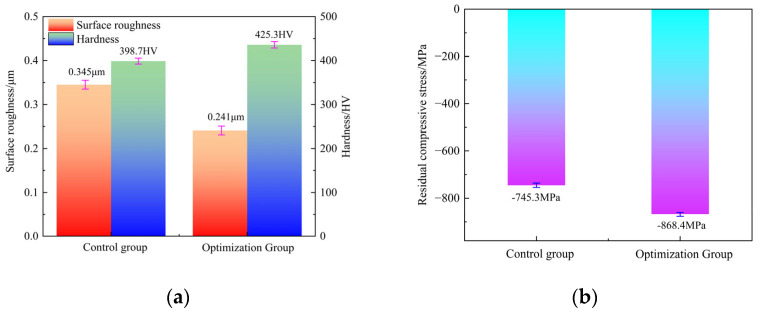
Comparison of surface performance metrics between the optimized and control groups: (**a**) *R_a_* and *H_v_*, and (**b**) *σ_res_*.

Figure 11 presents a quantitative comparison of surface performance indicators obtained under the optimized and control parameter sets. When using the optimized parameters, the *R_a_* decreased to 0.241 μm, representing a reduction of 30.14% relative to the control group value of 0.345 μm. Meanwhile, the *H_v_* and *σ_res_* increased to 425.3 HV and −868.4 MPa, respectively, corresponding to improvements of 6.67% and 16.52% compared with the control group values of 398.7 HV and −745.3 MPa. Furthermore, when compared with the predictions of the IWOA–RBF model, the relative errors for *R_a_*, *H_v_*, and *σ_res_* were 1.24%, 0.87%, and 1.43%, respectively. These results demonstrate that the proposed optimization framework, which integrates the IWOA–RBF predictive model with the MOGWO algorithm, provides an effective and reliable approach for USRP parameter optimization and yields substantial improvements in the surface performance of TC4 titanium alloy.

## 5. Conclusions

(1) To overcome the issue of the WOA getting trapped in local optima, an enhanced version was developed by incorporating optimal point set population initialization, a sinusoidal nonlinear convergence strategy, and an adaptive inertia-based position update strategy. On this basis, an IWOA–RBF prediction model was developed, with USRP parameters as inputs and *R_a_*, *H_v_*, and *σ_res_* as outputs. The proposed model was evaluated using multiple metrics to assess its predictive performance. The IWAO-RBF predictive model achieved RMSE values of 0.0060 μm (*R_a_*), 6.39 HV (*H_v_*), and 20.01 MPa (*σ_res_*) and MAPE values of 1.34%, 1.71%, and 2.92%. Compared with the RBF and WOA–RBF models, IWAO-RBF achieved higher R^2^ values of values of 0.9603, 0.9421, and 0.9294, indicating its superior fitting accuracy. The results confirm that the IWOA-RBF model effectively captures the complex, nonlinear relationships between the USRP parameters and the surface performance indicators of TC4 titanium alloy, demonstrating its potential as a reliable tool for surface quality prediction and optimization in the USRP.

(2) A multi-objective optimization strategy was designed by integrating the MOGWO algorithm with a CRITIC–TOPSIS decision-making framework to identify the optimal USRP parameters for TC4 titanium alloy. Experimental verification showed that the optimized process parameters reduced *R_a_* to 0.241 μm, increased *H_v_* to 425.3 HV, and enhanced *σ_res_* to −868.4 MPa. Compared to the optimal parameter set from orthogonal experiments, *R_a_* was reduced by 30.14%, and both *H_v_* and *σ_res_* showed improvements of 6.67% and 16.52%, respectively. The largest relative error between predicted and actual values was less than 1.43%, indicating the strong predictive reliability of the model. The findings confirm the robustness and efficiency of the proposed IWOA–RBF and MOGWO optimization framework for optimizing USRP parameters of TC4 titanium alloy. Future research will aim to extend the model by incorporating additional process variables and surface integrity descriptors, such as subsurface microstructural characteristics and fatigue performance, to enhance its applicability and generalizability.

## Figures and Tables

**Table 1:** Factor levels of the USRP parameters.

Factors	Levels				
	1	2	3	4	5
*n*/r·min^−1^	150	250	350	450	550
*f*/mm·r^−1^	0.10	0.15	0.20	0.25	0.30
*F*/N	250	350	450	550	650
*A*/μm	3	6	9	12	15

**Table 2:** Surface performance measurement results of the orthogonal USRP experiments.

No	Factors				*R_a_*/μm		*H_v_*/HV		*σ_res_*/MPa	
	*n*	*f*	*F*	*A*	Mean	StDev	Mean	StDev	Mean	StDev
1	1	1	1	1	0.306	0.0062	325.4	4.23	−758.2	10.56
2	1	2	2	2	0.337	0.0084	335.6	3.54	−744.6	9.35
3	1	3	3	3	0.352	0.0056	320.3	5.27	−784.5	10.47
4	1	4	4	4	0.376	0.0073	345.7	3.83	−728.1	12.12
5	1	5	5	5	0.425	0.0092	358.2	4.75	−685.4	9.68
6	2	1	2	3	0.295	0.0084	347.8	3.48	−712.3	10.21
7	2	2	3	4	0.328	0.0117	356.4	3.62	−698.2	11.72
8	2	3	4	5	0.346	0.0053	368.9	4.14	−668.5	13.23
9	2	4	5	1	0.321	0.0075	332.1	4.65	−735.6	12.86
10	2	5	1	2	0.375	0.0123	378.5	4.36	−722.4	14.12
11	3	1	3	5	0.291	0.0086	365.2	3.94	−658.9	13.66
12	3	2	4	1	0.302	0.0078	342.6	4.27	−685.2	12.43
13	3	3	5	2	0.315	0.0093	354.8	4.43	−672.8	11.57
14	3	4	1	3	0.345	0.0064	398.7	5.14	−745.3	9.64
15	3	5	2	4	0.368	0.0102	392.4	4.35	−695.7	11.72
16	4	1	4	2	0.310	0.0085	373.1	4.67	−632.4	12.38
17	4	2	5	3	0.324	0.0076	365.7	3.93	−615.8	8.63
18	4	3	1	4	0.332	0.0093	387.2	4.35	−695.4	13.44
19	4	4	2	5	0.357	0.0144	395.8	3.77	−625.9	12.89
20	4	5	3	1	0.389	0.0097	345.9	3.62	−652.7	14.23
21	5	1	5	4	0.315	0.0133	361.5	4.73	−508.5	13.65
22	5	2	1	5	0.334	0.0074	384.2	4.54	−532.6	12.96
23	5	3	2	1	0.342	0.0113	336.8	4.92	−615.4	9.62
24	5	4	3	2	0.362	0.0084	367.3	5.27	−574.2	10.46
25	5	5	4	3	0.398	0.0062	375.6	3.64	−555.8	13.88

**Table 3:** Performance comparison of prediction accuracy for different models.

Performance Metric	Evaluation Index	RBF Neural Network	WAO–RBF Neural Network	IWOA–RBF Neural Network
*R_a_*	RMSE (μm)	0.0237	0.0178	0.0060
	MAPE (%)	6.04	5.12	1.34
	R^2^	0.5743	0.7036	0.9603
*H_v_*	RMSE (HV)	12.32	9.36	6.39
	MAPE (%)	3.74%	2.84%	1.71%
	R^2^	0.4123	0.7698	0.9421
*σ_res_*	RMSE (MPa)	48.94	40.05	20.01
	MAPE (%)	6.19%	5.43%	2.92%
	R^2^	0.6502	0.6624	0.9294

**Table 4:** Comparison of 95% confidence and prediction intervals for model performance accuracy.

Performance Metric	Evaluation Index	RBF	WAO–RBF	IWOA–RBF
*R_a_*	95% CI	±0.0328	±0.0269	±0.00977
	95% PI	±0.0803	±0.0658	±0.0237
*H_v_*	95% CI	±17.23	±14.45	±10.44
	95% PI	±42.11	±36.27	±27.79
*σ_res_*	95% CI	±81.1	±62.5	±29.3
	95% PI	±198.8	±153.0	±71.7

**Table 5:** Statistical summary of the top-ranked solutions from 30 independent MOGWO optimization runs.

Statistics	*n*/r·min^−1^	*f*/mm·r^−1^	*F*/N	*A*/μm	*Ra*/μm	*H_v_*/HV	*σ_res_*/MPa
Mean	410.3	0.14	373.4	11.6	0.238	429.2	−880.9
StDev	3.81	0.006	4.68	0.27	0.0027	1.82	5.86

## Data Availability

All data generated or analyzed during this study are included in the present article.
